# Relationship between genetic risk and *p*‐tau concentration on cognitive performance in a multiethnic population from Peru

**DOI:** 10.1002/alz70856_103195

**Published:** 2025-12-26

**Authors:** Claudia Rivera‐Fernandez, Nilton Custodio, Rosa Montesinos, Andres Muñoz‐Najar, Dolly Reyes‐Dumeyer, Giuseppe Tosto, Marcio Soto‐Añari

**Affiliations:** ^1^ Universidad Tecnológica del Perú, Arequipa, Peru; ^2^ Unidad de Investigación de Deterioro Cognitivo y Prevención de Demencia, Instituto Peruano de Neurociencias, Lima, Lima, Peru; ^3^ Equilibria, Lima, Lima, Peru; ^4^ Unidad de Investigación y Docencia, Equilibria, Lima, Peru; ^5^ Universidad Catolica San Pablo, Arequipa, Peru; ^6^ Instituto de Bienestar socioemocional, Universidad del Desarrollo, Santiago, Santiago, Chile; ^7^ Columbia University Irving Medical Center, New York, NY, USA; ^8^ Universidad Católica San Pablo, Arequipa, Peru

## Abstract

**Background:**

The presence of APOE 4 is considered the main genetic risk factor for the development of dementia. Additionally, plasma *p*‐tau has been shown to be an important biomarker associated with Alzheimer's disease. However, these factors have been understudied in Latin American ethnic minorities, such as the Quechuas and Aymaras. Our objective was to analyze the relationship between genetic risk and *p*‐tau concentration on cognitive performance in a multiethnic population from Peru.

**Method:**

We extracted data from the Genetics of Alzheimer's Disease in Peruvian Populations (GAPP) study (Pandey et al., 2025). We included healthy cognitive controls and mild cognitive impairment (MCI) participants. A total of 432 older adults (Mean Age = 70.77, SD = 7.66) were assessed using the Rowland Universal Dementia Assessment Scale (RUDAS) for general mental status, the Selective Reminding Test (SRT) for delayed memory, Benton's visual working memory test, and the Rosen test for visuoconstruction. APOE genotyping and plasma sample collection for biomarker identification are detailed in Pandey et al. (2025).

**Result:**

We observed that 55.56% of the older adults were healthy controls (40% mestizo, 60% indigenous), while 44.44% had been diagnosed with MCI (40% mestizo, 60% indigenous). Regression analysis showed that participants carrying at least one APOE allele (E3/E4 or E4/E4) and with higher *p*‐tau217 concentrations performed worse on delayed recall in the SRT (*B* = ‐2.506; *p* < .015), but not on the RUDAS, Rosen test, or Benson test. This effect did not appear to be mediated by ethnicity. Further analyses differentiated by ethnicity showed that being indigenous was associated with better scores on the Rosen test (*B* = 0.608; *p* < .000), while being mestizo was associated with better performance on the Benton recognition test (*B* = ‐1.09; *p* < .000) and general cognition (*B* = ‐2.084; *p* < .016).

**Conclusion:**

The presence of APOE and *p*‐tau217 concentration modulate episodic memory performance, while ethnicity appears to influence visuospatial, visuoconstructive, and general cognitive processes.

